# 丙戊酸钠致髋关节置换术后获得性凝血因子XIII缺乏症1例报告及文献复习

**DOI:** 10.3760/cma.j.cn121090-20241130-00514

**Published:** 2024-12

**Authors:** 闽霞 张, 欢 白, 宁 唐, 振亚 洪, 伟凯 张

**Affiliations:** 1 华中科技大学同济医学院附属同济医院检验科，武汉 430030 Department of Clinical Laboratory, Tongji Hospital, Tongji Medical College, Huazhong University of Science and Technology, Wuhan 430030, China; 2 华中科技大学同济医学院附属同济医院血液内科，武汉 430030 Department of Hematology, Tongji Hospital, Tongji Medical College, Huazhong University of Science and Technology, Wuhan 430030, China; 3 华中科技大学同济医学院附属同济医院骨科，武汉 430030 Department of Orthopedics, Tongji Hospital, Tongji Medical College, Huazhong University of Science and Technology, Wuhan 430030, China

## Abstract

凝血因子XIII（FXIII）缺乏症是一种罕见疾病，且凝血常规实验并不能反映其缺乏而极易漏检。FXIII缺乏症又分为遗传性FXIII缺乏症和获得性FXIII缺乏症。对于FXIII缺乏症的诊断需要进行FXIII活性和抗原的检测，最终通过基因检测确诊。由于FXIII缺乏会导致纤维蛋白凝块不稳定，因此FXIII缺乏症的临床特点为脐带出血、软组织血肿、反复流产、脑出血和伤口愈合延迟等。多种原因可造成获得性FXIII缺乏症，本文介绍1例由于丙戊酸钠的长期使用导致在髋关节置换术后发生获得性FXIII缺乏症患者。

## Abstract (en)

Coagulation factor XIII（FXIII）deficiency is a rare disease that is easily overlooked because routine coagulation tests do not reflect its deficiency. FXIII deficiency is divided into hereditary FXIII deficiency and acquired FXIII deficiency. The diagnosis of FXIII deficiency requires testing for FXIII activity and antigen, with confirmation through genetic testing. Due to the instability of fibrin clots caused by FXIII deficiency, the clinical features of FXIII deficiency include umbilical cord bleeding, soft tissue hematoma, recurrent miscarriage, cerebral hemorrhage, and delayed wound healing. Acquired FXIII deficiency can be caused by various reasons, and this article introduces a case of acquired FXIII deficiency in a patient after hip replacement surgery due to long-term use of sodium valproate.

凝血因子XIII（FXIII）缺乏症分为遗传性和获得性，相比于遗传性FXIII缺乏症的罕见性，获得性FXIII缺乏症更为常见[1]。获得性FXIII缺乏症按病因分类，又分为免疫介导和非免疫介导。获得性FXIII缺乏症可由多种疾病、药物、手术等情况引起，造成患者伤口愈合不良、瘀斑、血肿等临床症状，严重者会引起颅内出血等危及生命的情况。

丙戊酸钠（VPA）属于丙戊酸类药物，是一种广谱抗癫痫药物。丙戊酸可致多种凝血功能异常，包括血小板减少症、血小板功能缺陷、低纤维蛋白原血症、获得性FXIII缺乏症及获得性血管性血友病[2]。本文报道了一例VPA致髋关节置换术后获得性FXIII缺乏症的病例，为此类患者治疗凝血功能障碍提供一定的参考。

## 病例资料

患者，女，25岁，脑瘫病史，长期服用VPA治疗，患者自诉半年前，右髋部着地，未进行处理，后因间断性感右下肢疼痛来我院门诊就诊，门诊X线提示右股骨骨折。2024年6月30日，患者以右股骨颈骨折诊断入院，准备进行骨科手术治疗。入院后完善检查，血常规：WBC 4.3×10^9^/L，RBC 4.31×10^12^/L，HGB 120 g/L，PLT 215×10^9^/L。肝肾功能正常，凝血常规中活化部分凝血活酶时间46.3 s（正常参考值25.0～38.0 s），其余正常。体格检查中患者体温、脉搏、呼吸均正常，血压为117/75 mmHg。患者无高血压、心脏病、肝病、结核等病史。患者于7月2日行右股骨颈骨折切开复位内固定术及髋关节置换术，手术顺利，术后返回病房。术后第1天，患者自诉伤口周围肿胀疼痛。同时患者血压降为85/47 mmHg，心率波动于98～120次/min。急查血常规：WBC 19.49×10^9^/L，中性粒细胞占86.2％，RBC 1.98×10^12^/L，HGB降至54 g/L。凝血常规：凝血酶原时间14.4 s，纤维蛋白原（FIB）1.9 g/L，D-二聚体定量（D-D）5.94 µg/mL FEU，其余均正常，详见表1。考虑患者术后出血，骨科病房停止术后常规抗凝治疗（普通肝素），并且给患者输注悬浮红细胞4 U。术后第2天，患者伤口疼痛并未改善，在对伤口处进行B超检查发现（图1）：右侧髋关节切口内侧肌层后方距体表约2.5 cm可见一范围6.4 cm×2.8 cm无回声区，内可见光点群及光带回声。其深部另可见宽度约3.0 cm的不规则无回声区（内可见杂乱光点群回声）似突破骨皮质，后方结构显示不清。考虑积血可能。同时血常规中HGB进一步下降到41 g/L。骨科病房考虑患者伤口愈合不良，存在持续性失血，输注悬浮红细胞治疗效果不佳，遂请血液科会诊，血液科医师建议进一步完善凝血因子活性、血小板功能、VWF及FXIII抗原检测。其中凝血因子的活性虽略微下降但并未降到一般止血水平以下，VWF活性及抗原均正常。其中FXIII抗原水平降低最为明显，只有9.8％。于是加做了氯乙酸溶解实验，患者血浆形成的纤维蛋白凝块在氯乙酸溶液中1 h内全部溶解，氯乙酸溶解试验阳性，提示患者血浆FXIII活性缺乏（<5％）。随后又加做了基于血栓弹力图（TEG）的测定FXIII抑制物的实验（图2），患者管与正常对照管相对差异大于10％，提示患者存在FXIII抑制物。根据ISTH的诊断标准，该患者拟诊断为获得性FXIII缺乏症。在排除了自身免疫性疾病如SLE、RA及恶性肿瘤、DIC等疾病后，血液科医师考虑造成患者获得性FXIII缺乏的主要原因为患者长期服用的VPA。于是术后第3天骨科联系血液科和神经内科进行会诊，会诊提出了以下治疗方案：①停VPA，换用奥卡西平片；②输注悬浮红细胞和冷沉淀，补充FXIII和纠正贫血；③密切监测患者血常规、凝血常规、FXIII抗原等指标。术后经过积极治疗，患者HGB、FXIII抗原及活性恢复（表1），伤口愈合良好并出院。

**表1:** 患者手术前后血常规及凝血常规检查结果

项目	术前	术后第2天	术后第3天	术后第4天	术后第8天	术后第10天	正常参考值
PT（s）	10.9	16.7	11.6	11.5	11.1	12.4	9.4～12.5
APTT（s）	46.3	39.1	30	24.2	39.6	45.5	25.0～38.0
FIB（g/L）	3.28	3.19	4.50	5.29	4.70	6.00	1.9～4.0
HGB（g/L）	120	41	73	72	86	89	115.0～150.0
PLT（×10^9^/L）	215	85	95	92	170	267	125.0～350.0
FIB（g/L）	3.16	1.75	4.50	5.29	\	\	2～4
D-二聚体（µg/ml FEU）	0.42	2.63	1.03	0.75	\	\	<0.5
FXIII抗原（％）	60.8	9.8	\	19.8	30.8	41.2	61～127
2％氯乙酸溶解试验	\	阳性	\	\	阴性	阴性	阴性
FXIII抑制物	\	阳性	\	\	阴性	阴性	阴性
FⅧ（％）		91					60～150
FⅨ（％）		65					60～150
FⅪ（％）		39					60～150
FⅡ（％）		48					70～120
FⅤ（％）		48					70～120
FⅦ（％）		61					55～170
FⅩ（％）		39					70～120

**图1:**
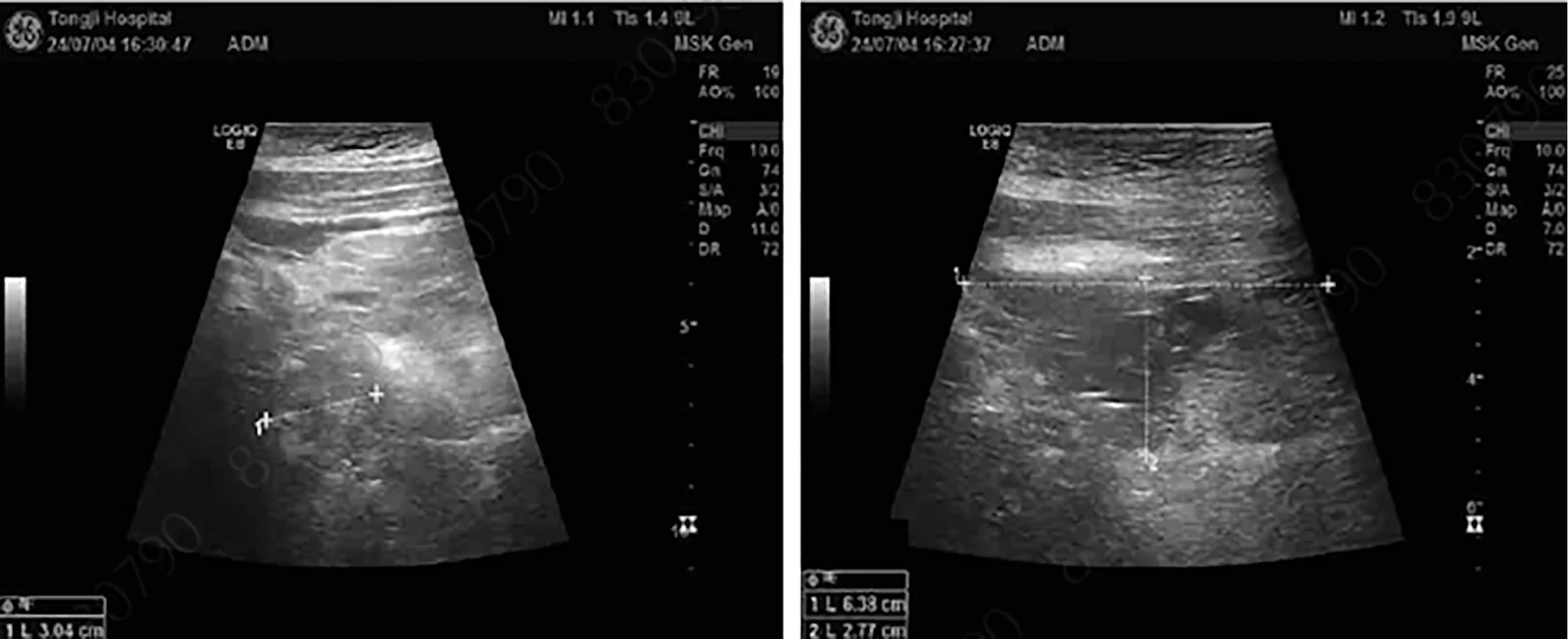
术后第2天患者伤口B超结果

**图2:**
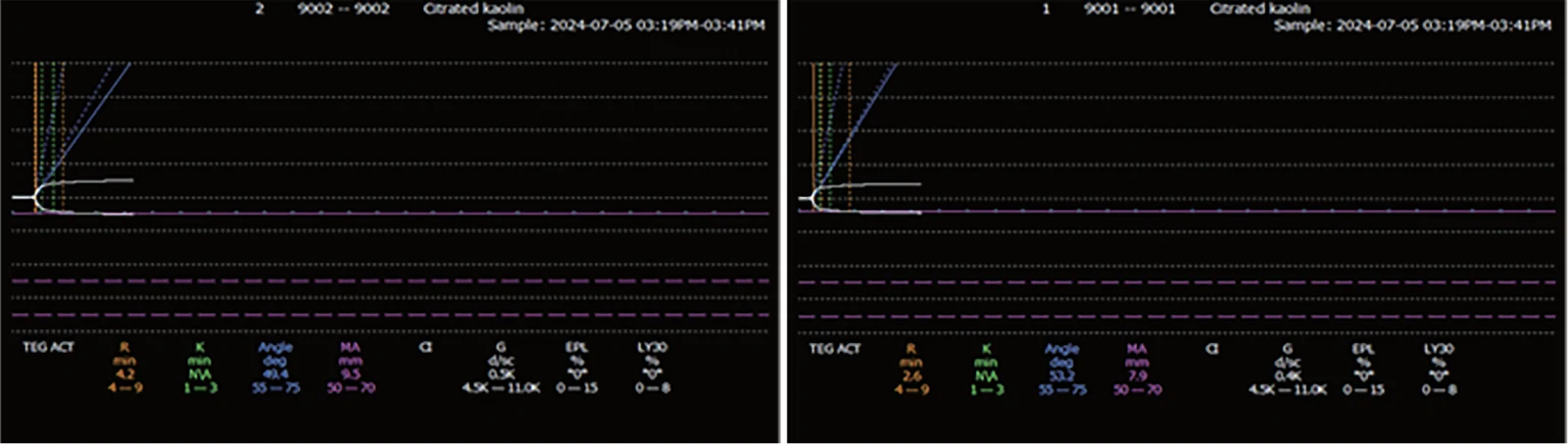
基于血栓弹力图（TEG）的测定XIII因子抑制物的实验 **A** 患者血清＋正常对照血浆患者血清+正常混合血浆1∶1混合后加入接触激活剂（如APTT试剂）、氯化钙，获得血浆TEG MA1结果（MA1=7.9）；**B** 正常血清+正常混合血浆1∶1混合后加入接触激活剂（如APTT试剂）、氯化钙，获得血浆TEG MA2结果（MA2=9.5）。（MA2-MA1）/MA2>10％说明存在FXIII抑制物（TEG-5000 MA参数声明不精密度为<10％）

## 讨论及文献复习

本文报道了一例VPA所致髋关节置换术后获得性FXIII缺乏症的病例，以期为此类患者凝血功能障碍的临床诊疗提供依据与参考。FXIII为参与凝血级联反应的最后一步，即在钙离子的参与下被凝血酶活化，A亚基与B亚基解离激活，被激活后的A亚基才是发挥活性的部分，它与纤维蛋白单体、纤维蛋白和α2-纤溶蛋白抑制剂交联，起到稳定纤维蛋白凝块和抵抗纤溶的作用。因此大部分FXIII缺乏的患者典型症状为自发性或延迟的术后出血[10]。FXIII缺乏不能通过常规凝血实验检出，因此对于临床上凝血常规无明显异常的出血患者，应检测FXIII活性等。根据ISTH的指南[9]，诊断FXIII缺乏症首先要进行FXIII活性实验，如ERICHROM®FXIII测定法、BIOPHEN®FXIII检测、荧光测定、氨释放测定/光度测定法、胺掺入测定及异肽酶测定等[1]。但国内暂无注册试剂，因此更多的是使用定性实验即尿素溶解实验或氯乙酸溶解实验进行检测。该方法的灵敏度取决于凝血因子和增溶剂等因素，虽然具有较好的特异性，灵敏度较差[11]。如本室使用的氯乙酸溶解实验的灵敏度为5％。但FXIII的一般止血水平为3％～10％，因此溶解实验阳性则可以说明FXIII活性不足以达到一般止血水平，可以判断为FXIII活性缺乏。再根据FXIII抗原结果对FXIII缺乏症进行分类，最后通过基因检测进行确诊。如果氯乙酸溶解实验阳性，本室还可以进一步检测FXIII抑制物，该方法是本室自建的基于TEG的平台检测FXIII抑制物的方法[13]，为确定FXIII缺乏症是免疫介导的还是非免疫介导的提供了实验依据。

VPA属于丙戊酸类药物，是一种广谱抗癫痫药物。丙戊酸是由天然存在的戊酸衍生而来的短链脂肪酸，与蛋白高度结合。它通过多种机制减少癫痫发作，如增加γ-氨基丁酸（GABA）合成、抑制GABA降解；减少兴奋性氨基酸β-羟基丁酸的释放；抑制兴奋性递质N-甲基-D-天冬氨酸（NMDA）的作用；阻断电压门控的钠离子通道；阻断钙离子通道等。丙戊酸主要由肝脏代谢，少量随尿液排出[3]。既往已有文献报道[8]，丙戊酸的应用可致多种凝血功能异常，包括血小板减少症、血小板功能缺陷、低纤维蛋白原血症、获得性FXIII缺乏症及获得性血管性血友病[6]。丙戊酸相关凝血异常的确切机制尚不明确，可能与以下几点相关：①免疫清除：丙戊酸会使机体产生针对血小板或其他凝血成分的自身抗体，造成血小板减少、凝血因子缺乏[4]–[5]；②合成减少：丙戊酸对骨髓抑制和药物肝毒性都会使血小板、凝血因子（如FXIII）合成减少[5]；③活性抑制：有研究表明丙戊酸会抑制血小板的环氧化酶通路和脂氧化酶通路，进而抑制血小板的活化和聚集[6]；④消耗增多：有研究表明丙戊酸可诱导组织型纤溶酶原激活物表达上调，造成纤维蛋白原降低[7]。Pohlmann等[8]报道了1例39岁的男性患者长期服用VPA，杏仁核海马切除术后出现严重颅内出血，FXIII活性仅17％，停VPA并使用FXIII浓缩物替代治疗后，FXIII水平逐渐恢复至正常。Teich等[9]报道了2例接受VPA治疗的儿童均表现为反复鼻出血症状，实验室检查显示FXIII缺乏伴血小板减少和血管性血友病因子（VWF）降低，减少或停止VPA治疗几日后，临床症状消失，实验室检查结果趋于正常。Pohlmann-Eden等[8]主持的一项前瞻性研究结果显示，接受VPA治疗的儿童中13％的患儿存在FXIII水平的降低[8]。以上研究均表明，VPA的使用与FXIII减少密切相关。值得关注的是，为何VPA的使用会导致FXIII减少，Gerstner等[6]提出猜想，丙戊酸进入人体后，作为外来物质，可诱导机体产生针对VPA的自身抗体，另由于VPA的结构与细胞膜脂肪酸的结构及化学成分相似，可诱导机体产生针对包括FXIII抗体在内的多种凝血成分自身抗体，最终导致机体凝血功能的异常[5]。Barr等[4]主持的一项前瞻性研究发现，近一半接受VPA治疗的患者血液中血小板相关抗体水平升高。停药后抗体滴度迅速降低[3]，印证了以上Gerstner等提出的猜想。本例患者术后FXIII缺乏且产生FXIII抑制物，并且停药后FXIII抑制物转为阴性，FXIII活性恢复正常，均提示VPA可导致机体的免疫功能失调，另手术可致患者内环境紊乱、免疫功能失调，患者在药物和手术的双重打击下，产生一过性自身抗体，造成FXIII缺乏，引起术后伤口血肿。有趣的是，Kumar等[2]的一项回顾性研究发现使用丙戊酸的患者在进行骨科手术时出血量较使用其他抗癫痫药的患者多，但在接受神经外科手术的患者中并未观察到类似出血量增加的现象。笔者猜想这可能与神经外科手术和骨科手术在创面大小、手术方式等方面的差异相关，另亦可能与骨科手术患者需长期服用VPA，而神经外科患者仅术后预防性服用VPA，两者在剂量、耐受性等方面的差异相关。本例与Kumar等[2]报道的这一有趣现象吻合，从侧面印证患者术后FXIII缺乏与丙戊酸的长期使用相关。

针对丙戊酸导致FXIII缺乏的治疗目前无相应的指南和专家共识，但根据以往的案例报道及临床经验，在丙戊酸使用期间应密切监测FXIII，若存在出血表现或倾向应减量或停用VPA，出血严重者需进行包括冷沉淀、FXIII浓缩剂在内的替代治疗[2],[8]–[9]。

VPA相关获得性FXIII缺乏症的研究报道仍较少见，我们在此报道了1例VPA所致髋关节置换术后获得性FXIII缺乏症的病例，结合临床及实验室表现，以期为此类患者凝血功能障碍的精准诊疗提供依据与参考。
